# A Quantitative and Qualitative Analysis of the Patient and Caregiver’s Perspective on Outcomes of Intravenous Administration of Low-Dose Ketamine for C-PTSD, TBI, and Treatment Resistant MDD: A Clinical Example

**DOI:** 10.3390/reports8010013

**Published:** 2025-01-22

**Authors:** Laura Hentig, James Hentig, Jessica M. Gill

**Affiliations:** 1 Department of Health Sciences, University of Colorado-Colorado Springs, Colorado Springs, CO 80918, USA; lhentig@uccs.edu; 2 School of Nursing, Johns Hopkins University, Baltimore, MD 21205, USA; jessicagill@jhu.edu; 3 School of Public Health, Brown University, Providence, RI 02912, USA

**Keywords:** ketamine therapy, treatment-resistant depression TRD, major depressive disorder MDD, complex post-traumatic stress disorder C-PTSD, traumatic brain injury TBI

## Abstract

**Background and Clinical Significance**: Treatment resistant psychiatric disorders affect millions of people across the globe. Ketamine has been employed as a treatment option for those with treatment resistant depression, as well as for chronic pain and alcohol use disorder. However, case presentations and research has been limited on the outcomes, and furthermore there is even less on the patient or caregiver perspective on the impact of the treatment. **Case Presentation**: Here, we present a middle-aged male who has undergone 20 intravenous (IV) ketamine infusions to treat Complex Post-Traumatic Stress Disorder (C-PTSD) and Major Depressive Disorder (MDD). We provide both qualitative perspectives from the patient, caregiver, as well as quantitative analyses of the patient from the Patient Health Questionnaire-9 (PHQ9), Beck’s Depression Inventory (BDI), and the Neurobehavioral Symptom Inventory (NSI) following treatment and weekly (4 weeks) assessments between treatments. **Conclusions**: Collectively, these data provide a holistic view of the use of ketamine for this patient on a variety of mental health, physiological, and behavioral conditions.

## 1. Introduction and Clinical Significance

Traumatic brain injuries (TBI) are an injury to which everyone is susceptible, and the injury is capable of causing significant disability following all ranges of severity [1]. Mild TBI (mTBI) is among the most common, accounting for 70–90% of all reported TBIs affecting ~2 million people in the US each year [2]. Although many individuals rapidly recover from associated sequelae within 1–3 months, which can range from dizziness, headaches, and sensitivity to light and sound, major depressive disorder is a known disorder correlated with the injury that can have prolonged effects for TBI survivors [3,4,5].

Major depressive disorder (MDD) is yet another debilitating disorder, which meets the definition set by the *DSM-5* if consistent depressed mood, anhedonia, or hopelessness persists for 2 or more weeks (observed or self-reported), in conjunction with meeting at least 5 of the 9 major depression criteria. In the US, there is a prevalence of MDD of ~20% with higher lifetime prevalence in women (26.1%) than in men (14.7%) [6]. However, MDD is a commonly reported psychiatric condition associated with TBI [5]. For TBI survivors, prevalence rates have been reported to be 33% higher than uninjured matched controls, rising above 50% one-year post-injury, and they have a 2-fold increased likelihood of suicide [7,8,9]. Exacerbating this issue, the prevalence rate of treatment-resistant depression is ~30% of those diagnosed with MDD. Although there is not a true consensus on the definition of treatment-resistant depression (TRD), the authors acknowledge the colloquial definition of failure to respond to two or more pharmacological therapies each administered for greater than 2 months, and an estimated 30% of individuals with MDD exhibit TRD [10]. As traditional pharmacological treatments have struggled, recent efforts have begun to explore the potential psychedelics such as psilocybin, 3,4-Methylenedioxymethamphetamine (MDMA), and off-label use of ketamine [11,12,13].

Ketamine, an N-methyl-D-aspartate (NMDA) receptor antagonist initially synthesized by organic chemist Calvin Stevens in 1962, was originally developed as an anesthetic [14]. The drug produces a “dissociative anesthesia” keeping airways intact. A growing body of the literature has demonstrated the clinical value of ketamine across diverse settings, with emerging roles in pain medicine and emerging data that suggest it has a strong efficacy against TRD for individuals with MDD [13]. Along with IV ketamine, the enantiomer S-ketamine or esketamine, has become a popular treatment option that is delivered through nasal inhalation on a daily dose schedule. While there is convenience in esketamine, the literature suggests that IV ketamine has greater efficacy, greater remission rates, and has greater compliance [15]. Nevertheless, there is not full concordance with what route, rate, or dose of ketamine may be the most efficacious [16].

Here, we describe the clinical presentation of a TBI survivor with various psychiatric conditions who has undergone 1.5 years of regular IV ketamine treatments (20 treatments). We provide qualitative insights, through patient and caregiver perspectives, as well as quantitative overviews of the impact of IV ketamine on the patient’s psychological wellbeing (PHQ9, BDI), and neurobehavioral symptoms across cognitive, somatic, and affective subscales (NSI) spanning from 1 to 4 weeks post-ketamine treatment.

## 2. Case Presentation

### 2.1. Case Presentation

A 34-year-old male and disabled combat veteran diagnosed with a multiple mild traumatic brain injury (first diagnosed TBI in 2010 via MACE and OSU-TBI-ID, service connected), complex-post-traumatic stress disorder (PTSD, diagnosed in 2011 via DSM-IV, service connected), chronic pain (diagnosed in 2011), and major depression disorder (diagnosed in 2011 via DSM-IV, service connected) sought treatment for TRD through intravenous (IV) ketamine therapy. The patient had previously tried various medication classes including: antimanics, antipsychotics, anxiolytics, benzodiazepines, mood stabilizers, norepinephrine–dopamine reuptake inhibitors (NDRI), selective serotonin and norepinephrine reuptake inhibitors (SSNRI), selective serotonin reuptake inhibitors (SSRI), tetracyclic antidepressants (TeCA), and tricyclic antidepressant (TCA) (Table 1). Additionally, the patient underwent group psychotherapy, individual psychotherapy, prolonged exposure psychotherapy, and eye movement desensitization and reprocessing (EMDR) with self-reported minimal relief of depressive symptoms.

**Figure 1:**
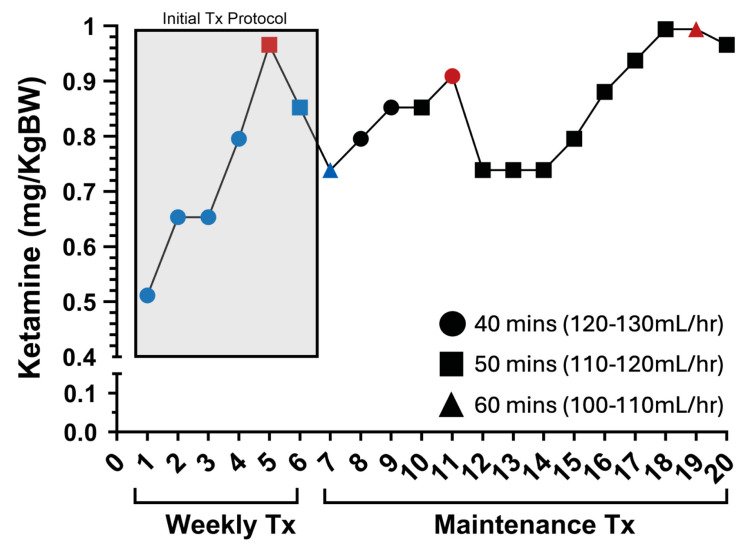
**Dose Schedule.** Graphical representation of the subject’s ketamine dose by visit, accompanied whether the infusion began with a 5 mg bolus, the total infusion time/run rate, and whether the treatment was disrupted. Grey box; initial six treatments with a protocol of one treatment a week, blue = with bolus, black = without bolus, red = disrupted treatment.

The patient entered treatment with increased suicidality and underwent an initial 6 week treatment of IV ketamine. The standard of care for the facility was 1–2 treatments weekly for the first 4–6 weeks. The patient, due to logistical ease and to reduce financial burden, received one treatment weekly, for six weeks, starting at 0.5 mg/kg of body weight at session one and progressively increased the dose over the first six sessions to 1 mg/kg of body weight delivered at a flow rate of (1.6 mL/min) (Figure 1, grey box). Due to the patient’s anxiety surrounding the treatment, propensity to experience panic attacks during the infusion, and combativeness, Versed was administered 5 min prior to beginning the ketamine infusion. The patient then began maintenance sessions occurring every 2–4 weeks and underwent 20 IV infusions over 1.5 years at reduced doses ranging (0.5–0.99 mg/kg of bodyweight, Figure 1). After each treatment the patient took extensive notes immediately after (within 30 min of completion of dose) followed by reflections the following days and weeks later regarding their experience and mood. Concurrently, he attended individual psychotherapy and coadministration of a SNRI (duloxetine; 30–60 mg) for 6 months, which was then discontinued for a mood stabilizer (lamotrigine; 100–300 mg) for 6 months, before being discontinued for an antipsychotic (current therapy, aripiprazole; 2 mg).

### 2.2. Quantitative Scales

**Figure 2:**
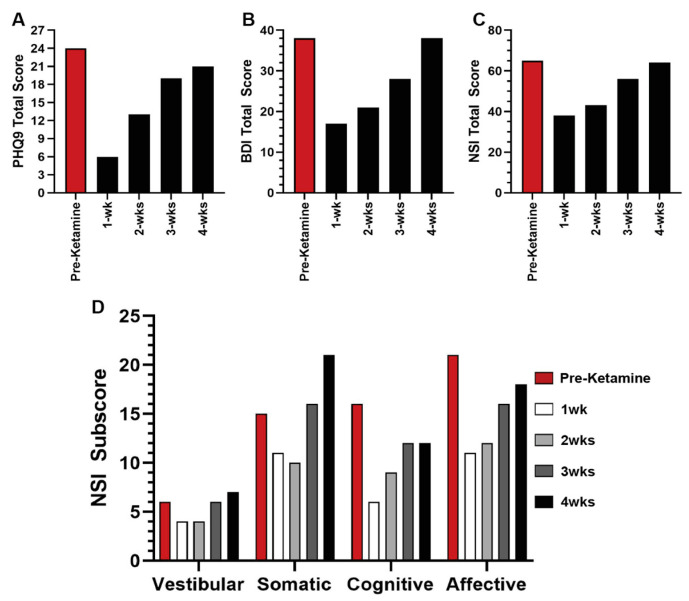
**Quantitative Scales.** (**A**–**C**) Rapid reduction in (**A**) PHQ9, (**B**) BDI, and (**C**) NSI total score was observed with progressive return near baseline by 4 weeks post infusion. (**D**) NSI-sub scores displayed similar trends over 4 weeks with somatic scores exceeding baseline.

The subject experienced profound relief in symptomology following ketamine treatment; however, the effect decreased as the time from the treatment increased. In the initial 1- and 2 weeks post-ketamine treatment, the patient reported substantially decreased PHQ9, BDI, and NSI total score and sub scores (Figure 2A–D). However, by 3 weeks post-treatment, the patient reported symptom scores approximately 75% of, or neared, pre-ketamine reports. By 4 weeks post-treatment, the patient reported scores at, or exceeding, initial baseline scores for PHQ9, BDI, and NSI (Figure 2A–D). Interestingly, the patient’s somatic subscore worsened 4 weeks post-ketamine, exceeding the pre-ketamine levels (Figure 2D).

### 2.3. Patient’s Experience and Perception

Following the first initial treatments in which an escalated dose plan was followed, the patient reported better outcomes with lower doses and the patient reported immediate psychiatric relief with decreasing suicidal ideation. The patient wrote after their first treatment (0.5 mg/kg body weight), “I went into the session prepared to commit suicide days later, however, I left raw, and clear headed. Things were not better, but I felt more okay with the issues. I felt okay talking about things I have never told anyone”. During the fifth treatment (0.97 mg/kg body weight) the patient experienced an adverse event, completely dissociated, and experienced extreme panic in which the treatment was discontinued prematurely and 1 mg of Versed was administered. The patient was in distress, combative, and unaware of person or place. The patient recalls this event writing, “I came out in shear panic, surrounded by [my physician and wife], not knowing what was real, or more importantly, what happened to cause them to pull me out”. Although the patient reports having experienced both good and stressful experiences, he also reports that regardless of the treatment experience, the ketamine treatment results in relief from depressive episodes and reduced suicidality.

### 2.4. Caregiver’s Experience and Perception

The caregiver, a 34-year-old female, who is his spouse and a medical professional, was the one to suggest ketamine therapy after seeing the subject experience such deep depressive states. The caregiver stated, “Prior to starting the treatments, his days were often clouded by emotional outbursts, a pervasive sense of sadness, and reoccurring suicidal ideation. He would often retreat to his bed, overwhelmed by a desire to escape the world”.

The caregiver attended every session and participated in pre-treatment evaluation of the patient’s response to the previous treatment. She shared her perception of the treatment stating, “The effects of the treatment were profound. While under its influence, he described vivid images and sensations, which I diligently recorded for him so he would write in his diary for future reference. Upon regaining consciousness, there was a noticeable shift in his demeanor. The heavy burden of sadness seemed to lift, replaced by a newfound sense of lightness. In the hours and days following the treatment, his mood continued to improve. He no longer felt weighed down by the darkness of depression, instead, expressing a renewed interest in engaging with the world around him. Simple activities like hiking and engaging in conversations became enjoyable once again. The positive effects of the treatment persisted for several weeks, during which time he was able to maintain good mental health and hygiene”. However, she described the effects of the treatment as transient, “However, as the weeks passed between treatments, I began to notice a change in his mindset. Agitation crept in, signaling the impending return of his depression”.

Nevertheless, the caregiver finished her interview with the following, saying, “Despite the decline in effect over weeks from the treatment, the ketamine treatments have provided invaluable relief for my husband, allowing him to function as a healthy adult and giving me peace of mind regarding his well-being. With regular visits spaced approximately 4–5 weeks apart, we have been able to maintain a resemblance of normalcy in our lives, albeit with the knowledge that the battle against depression is ongoing”.

## 3. Discussion

Here, we provide an account of the use of IV ketamine in the coadministration of psychotherapy and psychopharmacological treatments for a male patient with a set of complex psycho-comorbidities. Both the patient and the caregiver convey the treatment as positive and effective, and their perceptions of the lasting effects of the treatment align with the quantitative measurements of psychiatric wellbeing through the PHQ9, BDI, and NSI. The patient is a responder to IV ketamine, with dramatic improvements in psychiatric wellbeing; however, the patient’s mental health rapidly deteriorates and returns to baseline on most measurements by 4 weeks post-treatment.

Although some reports suggest individuals may respond to ketamine with longer bouts of remission following initial treatments, a subset of individuals, like our patient, may require a maintenance schedule consisting of ketamine treatment every 3–6 weeks to maintain a response and a reduction in depressive symptoms [17]. However, prolonged use of ketamine comes not without potential complications, with cognitive decline and memory impairment the primary concerns. Although we have no quantitative assessments of the patient’s cognitive abilities over the course of his treatment, it is worth noting that the patient started and finished an advanced degree with a 4.0 GPA during the course of his ketamine treatments. Examining a subset population of patients using chronic treatment of ketamine for TRD ranging from 12 to 46 treatments, Wilkinson and colleagues [17] found no evidence of cognitive decline using multiple exams testing attention, working memory, visual memory, verbal memory, and short- and long-term recall.

Interestingly, we saw a similar reduction in the patient’s NSI subscale scores, which largely followed a similar pattern of rapid reductions with a progressive return of pre-ketamine scores, with the exception of the NSI somatic subscale. This subscale, which includes headache, nausea, vision problems, light sensitivity, noise sensitivity, numbness/tingling, and taste/smell changes, rose above pre-ketamine levels 4 weeks post-ketamine. Although not reported here, the BDI also has subscales which include a somatic subscale. When assessed, we saw similar rapid reductions and a progressive return, and similarly we observed the BDI somatic subscale slightly higher at week four than pre-ketamine scores. Ketamine has been shown to have a significant impact on somatic sensory responses and is regularly used to pain [18], which is shown in the immediate effects; however, much like with depressive symptoms, this patient does not receive prolonged effects in pain relief either.

## 4. Conclusions

Although this patient represents a single observation, their experiences fall in line with previous reports [19]. However, to our knowledge no report includes the patient and caregiver perspective, particularly providing both qualitative and quantitative evidence behind the efficacy of the treatment and the duration of the refractory period experienced. This report adds to the building body of literature that suggests that IV ketamine can be a strong psychiatric tool, although some individuals may have a limited response.

## Figures and Tables

**Table 1:** Previous pharmacological interventions.

Drug Class	Drug Name	Effect
Antimanic	Lithium	Increased suicidial ideation
Antipsychotic	Aripiprazole	Effective on mood and dissociative episodes; severe weight gain
Anxiolytic	Buspirone	No effect
Benzodiazepin	Alprazolam	Effective rescue medication
	Clonazepam	Significant sedation and impaired cognition
Mood Stabilizer	Lamotrigine	Limited effect at lower dose; increased anxiety and paranoia at higher doses
NDRI	Bupropion	No effect
SSNRI	Duloxetine	Significant effect on mood; severe GI issues
	Venlafaxine	Significant effect on mood; severe weight gain
SSRI	Citalopram	No effect at multiple doses
	Escitalopram	No effect at multiple doses
	Fluvoxamine	No effect at multiple doses
	Paroxetine	No effect at multiple doses
	Sertraline	Mild effect on mood; severe weight gain
TeCA	Mirtazapine	No effect
TCA	Amitrptyline	Drowsiness; no other effect
Other	Prazosin (alpha blocker)	Effective for night terrors; led to low bp/additional TBI event
	Propranolol (beta blocker)	No effect (anxiety)

## Data Availability

The original contributions presented in this study are included in the article. Further inquiries can be directed to the corresponding author.
